# Monitoring of oral health teams after National Primary Care Policy 2017

**DOI:** 10.11606/s1518-8787.2020054002075

**Published:** 2020-10-28

**Authors:** Edson Hilan Gomes de Lucena, Carolina Dantas Rocha Xavier de Lucena, Josiane Aparecida de Souza Alemán, Gilberto Alfredo Pucca, Antônio Carlos Pereira, Yuri Wanderley Cavalcanti

**Affiliations:** I Universidade Federal da Paraíba Centro de Ciências da Saúde Departamento de Clínica e Odontologia Social João PessoaPB Brasil Universidade Federal da Paraíba . Centro de Ciências da Saúde . Departamento de Clínica e Odontologia Social . João Pessoa , PB , Brasil; II Fundação Oswaldo Cruz Instituto Aggeu Magalhães Programa de Pós-Graduação em Saúde Pública PernambucoPE Brasil Fundação Oswaldo Cruz . Instituto Aggeu Magalhães . Programa de Pós-Graduação em Saúde Pública . Pernambuco , PE , Brasil; III Universidade Federal da Paraíba Centro de Ciências da Saúde Núcleo de Estudo e Pesquisas Interdisciplinares em Biomateriais João PessoaPB Brasil Universidade Federal da Paraíba . Centro de Ciências da Saúde . Núcleo de Estudo e Pesquisas Interdisciplinares em Biomateriais . João Pessoa , PB , Brasil; IV Universidade de Brasília Faculdade de Ciências da Saúde Departamento de Odontologia BrasíliaDF Brasil Universidade de Brasília . Faculdade de Ciências da Saúde . Departamento de Odontologia . Brasília , DF , Brasil; V Universidade Estadual de Campinas Faculdade de Odontologia de Piracicaba Departamento de Odontologia Social CampinasSP Brasil Universidade Estadual de Campinas . Faculdade de Odontologia de Piracicaba . Departamento de Odontologia Social . Campinas , SP , Brasil

**Keywords:** Dental Health Services, Patient Care Team, Family Health Strategy, Health Status Disparities

## Abstract

**OBJECTIVE:**

To monitor the number of oral health teams implemented in the Family Health Strategy after National Primary Care Policy 2017.

**METHODS:**

This is a study of quantitative, descriptive and analytical nature that used the data from the public reports of the history of oral health coverage available in the e-Manager platform of Primary Care of the Ministry of Health of all Brazilian municipalities (5,570). The survival rate of the municipalities that did not reduce the number of oral health teams was analyzed according to the region of the country, human development index, Gini inequality index and population size. Cox regression was used to analyze the factors associated with the decrease in the number of teams implanted after 1, 3, 6, 9, 12, 15, 18 and 21 months of publication of the 2017 national policy ordinance, considering the hazard ratio (HR) and p < 0.05.

**RESULTS:**

After 21 months of publication of the policy, 6.7% of Brazilian municipalities reduced the number of oral health teams. This reduction was higher in the South (6.7%) and Northeast (4.8%), in municipalities with the highest human development index, i.e., greater than or equal to 0.7 (5.6%), more unequal in terms of income distribution (Gini index > 0.62) and larger population size (more than 100,000 inhabitants). Municipalities in the Northeast (HR = 1.220) and South (HR = 1.771) regions had a higher chance of reducing the number of teams compared with those in the North region. More unequal municipalities (HR = 6.405) and with larger population size (HR = 4.273) were also more likely to reduce the coverage of oral health teams.

**CONCLUSION:**

The municipalities that reduced the number of oral health teams in the Family Health Strategy are from the South and Northeast regions, with greater social inequality and larger population size. This scenario can significantly affect the population’s access to dental health services in the Unified Health System, especially among those in need.

## INTRODUCTION

Primary health care (PHC) is the level of care that develops a set of actions ranging from health promotion, disease prevention, protection, diagnosis and treatment, as well as rehabilitation, harm reduction, palliative care and health surveillance. These actions are directed to the population under the sanitary responsibility of health teams in a given territory, being developed in the individual, family and collective spheres ^1^ .

In Brazil, policies to strengthen PHC are essential for the implementation of the principles and guidelines of the Unified Health System (SUS) ^2^ . The organization of PHC in SUS differ from other models proposed in several countries due to three important characteristics: a multidisciplinary team responsible for certain geographic territories with the population enrolled, presence of community health agents and inclusion of the integral supply of oral health in the public health system ^3^ .

Historically, the insertion of dental practices in SUS occurred in parallel to the organization of other health services, offering actions centered on maternal and child and schoolchildren care, and the preventive focus was restricted to this population group. The mutilating profile of dental health actions developed up to the beginning of the 21st century resulted in a high prevalence of toothless individuals, which stimulated the transformation of dental health practices ^4^ . Although optionally, the incorporation of the oral health team (OHT) nationwide occurred eight years after the creation of the Family Health Strategy (FHS), in an attempt to reorganize the model of dental health care provision in PHC ^5^ . The inclusion of dental health in the FHS sought to break with the excluding, technical and biological dental practice, thus constituting an opportunity for change in the work process ^6^ .

The *Política Nacional de Saúde Bucal* (PNSB – National Oral Health Policy), *Brasil Sorridente* (Smiling Brazil), implemented in 2004, established guidelines in compliance with the foundations of SUS that reinforce the need to reorganize oral health care at all levels of care. In the scope of care, PNSB points fundamentally to the articulation of the oral health network to seek the integrality of care ^7^ .

Based on *Brasil Sorridente* , policies were implemented aimed at the expansion and qualification of oral health in PHC, such as adjustments in financial transfers, updating the rule for the implementation of OHT, matching the amount of OHT accredited to family health teams, inclusion of new clinical procedures in PHC and creation of the plan for providing dental equipment for each OHT that the municipality implement. In 2012, the redefinition of the composition of OHT within FHS was one of the strategies for consolidating oral health in PHC ^8 , 9^ .

Investments in *Brasil Sorridente* allowed an expansion of approximately 500% in the number of OHT, which allowed the expansion of 4,261 OHT in 2002 to more than 25,000 OHT in 2017, resulting in coverage of about 40% of the Brazilian population ^1^ . Despite the progress in more than 10 years of *Brasil Sorridente* , economic and political scenario has undergone transformations in recent years. The publication of Constitutional Amendment No. 95, which limited the ceiling on health and education spending, will have a significant long-term impact on the total investments destined to the sector, out of step with the demands of the population ^10^ . Decree no. 2,436 of the Brazilian Ministry of Health, which reformulated the *Política Nacional de Atenção Básica* (PNAB – National Primary Care Policy) in 2017, provides for the non-mandatory oral health in the Family Health Strategy ^11^ . The risk of possible setbacks, the disassistance of a significant part of the population and loss in the quality of PHC services after the publication of this decree was pointed out in the literature ^2 , 12^ .

Thus, our study sought to monitor the number of oral health teams implemented in the Family Health Strategy after the National Primary Care Policy 2017. This article analyzes the trend of the number of teams deployed and seeks to verify which factors are associated with the reduction in the number of OHT in Brazil between October 2017 and July 2019.

## METHODS

A study of quantitative, descriptive and analytical nature that used, from October 2017 to July 2019, from the public reports of the history of oral health coverage available in the e-Gestor platform of Primary Care of the Ministry of Health of all Brazilian municipalities.

The dependent variable (variation in the number of OHT implanted) was categorized as follows: “did not change or enlarged” and “reduced” the number of teams created. The independent variables of the study were: time – 1, 3, 6, 9, 12, 15, 18 and 21 months after the publication of the new PNAB; region – North, Northeast, Southeast, South and Midwest; human development index (HDI) – low (< 0.7) and high (≥ 0.7); Gini index – less unequal (≤ 0.61) and more unequal (> 0.62); and population size – up to 30,000 inhabitants, 30,001 to 50,000 inhabitants, 50,001 to 100,000 inhabitants and more than 100,000 inhabitants. The HDI and the Gini index were obtained from the 2010 Demographic Census, released by the United Nations Development Programme (UNDP). For this reason, the total number of municipalities for the variables HDI and Gini is 5,565, corresponding to the number of municipalities existing in 2010. The population size was obtained from the Brazilian Institute of Geography and Statistics (IBGE).

The data were initially analyzed by descriptive statistics to characterize the sample, and the absolute and percentage distributions were obtained. Then a bivariate analysis was performed by Pearson’s chi-square test to identify associations between the dependent and independent variables.

For the multivariate analysis, we estimated the cumulative survival of Brazilian municipalities that did not reduce, i.e., maintained or increased the number of OHT implanted. We used cox regression to analyze the factors associated with the reduction in the number of OHT implanted after 1, 3, 6, 9, 12, 15, 18 and 21 months of publication of the new PNAB ordinance. The factors region, HDI, Gini index and population size were used as independent variables that could predict the reduction in the number of OHT implanted. In the regression analysis, we used the variables HDI and Gini as continuous quantitative, without categorization.

Hazard ratio (HR) values were obtained for each category of associated factors, considering a 95% confidence interval and a 5% statistical significance. All tabulations and data analysis were performed in the Statistical Package for Social Sciences software (IBM-SPSS, v.24, IBM, Chicago, IL).

## RESULTS

**Figure 1:**
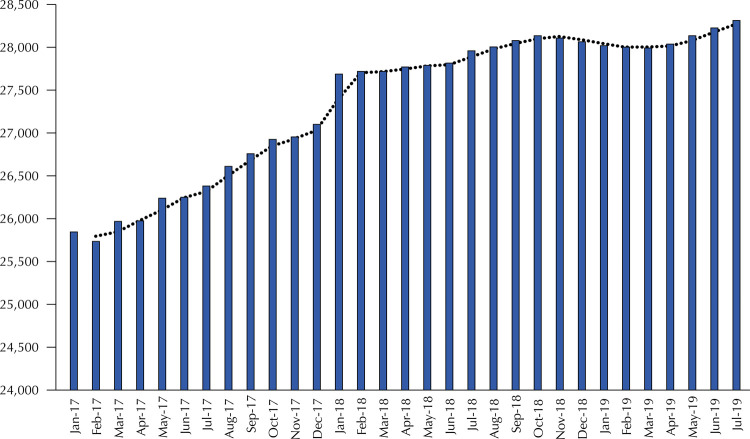
Number of oral health teams of the Family Health Strategy in Brazil between January 2017 and July 2019.

From January 2017 to July 2019, the number of oral health teams created in Brazil went from 25,848 to 28,311. However, the trend line indicates a stabilization in the quantity of these teams after January 2018, thus indicating a loss in the OHT creation amplitude ( Figure 1 ).

Jan: January; Feb: February; Mar: March; Apr: April; May: May; Jun: June; Jul: July; Aug: August; Sep: September; Oct: October; Nov: November; Dec: December

Between the 1st and 21st month of publication of the PNAB 2017 ordinance, the number of municipalities that reduced the number of OHT in the FHS tripled. This reduction was higher in the South (6.7%) and Northeast (4.8%), in municipalities with higher HDI (5.6%), more unequal in terms of income distribution (Gini index > 0.62) and larger population size (more than 100,000 inhabitants). All variables presented statistical significance in the bivariate model ( Table 1 ). However, there was no statistically significant difference (p = 0.081) between the number of municipalities that did not change or enlarge and those that reduced the number of OHT in the 18th and 21st month, which means that the number of municipalities that lost teams do not tend to reduce ( Table 1 ).

**Table 1:** Distribution of Brazilian municipalities according to the variation in the number of oral health teams (OHT) implemented, according to the time since the publication of the ordinance of the new National Policy of Primary Care, the country’s region, human development index (HDI), Gini index and population size.

		Variation in the number of OHT created				p
		Did not reduce		Reduced		
		n	%	n	%	
Time	1 month	5,457	98.0	113	2.0	< 0.001
	3 months	5,398	96.9	172	3.1	
	6 months	5,322	95.5	248	4.5	
	9 months	5,333	95.7	237	4.3	
	12 months	5,296	95.1	274	4.9	
	15 Months	5,187	93.1	383	6.9	
	18 Months	5,156	92.6	414	7.4	
	21 Months	5,203	93.4	367	6.6	
Region	North	430	95.5	20	4.5	< 0.001
	Northeast	1,708	95.2	86	4.8	
	Southeast	1,595	95.6	73	4.4	
	South	1,111	93.3	80	6.7	
	Midwest	450	96.4	17	3.6	
HDI	Low	3,502	96.4	130	3.6	< 0.001
	High	1,826	94.4	107	5.6	
Gini Index	Less unequal	5,133	95.8	225	4.2	0.010
	More unequal	195	94.3	12	5.7	
Population size	≥ 30,000 inhabitants	4,251	96.4	160	3.6	< 0.001
	30,001 to 50,000 inhabitants	458	92.9	35	7.1	
	50,001 to 100,000 inhabitants	319	91.4	30	8.6	
	More than 100,000 inhabitants	266	83.8	52	16.2	

**Figure 2:**
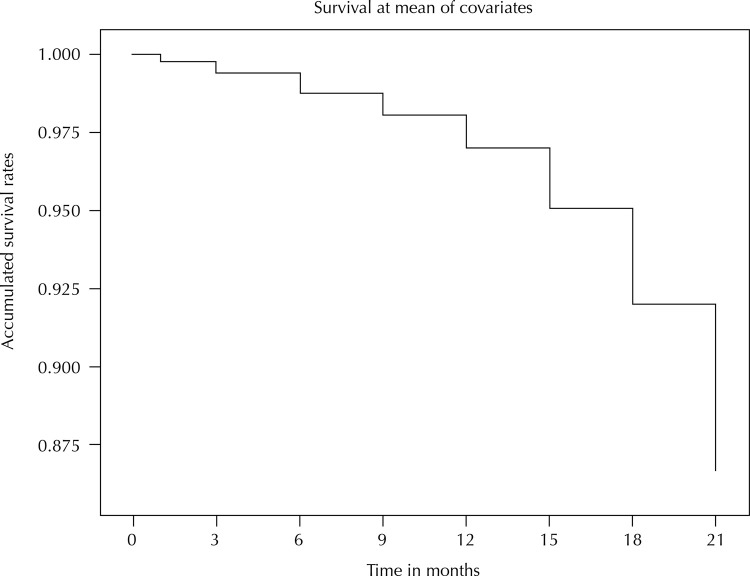
Accumulated survival rate of Brazilian municipalities that did not reduce the number of oral health teams between October 2017 and July 2019.

We found that 6.6% (n = 367) of Brazilian municipalities reduced the number of OHT in the Family Health Strategy 15 months after the publication of the PNAB in 2017. The survival curve of municipalities that did not reduce the number of OHT showed a greater reduction between November 2018 and July 2019 ( Figure 2 ). The greatest reduction was observed in April 2019 (18th month).

HDI: human development index

**Figure 3:**
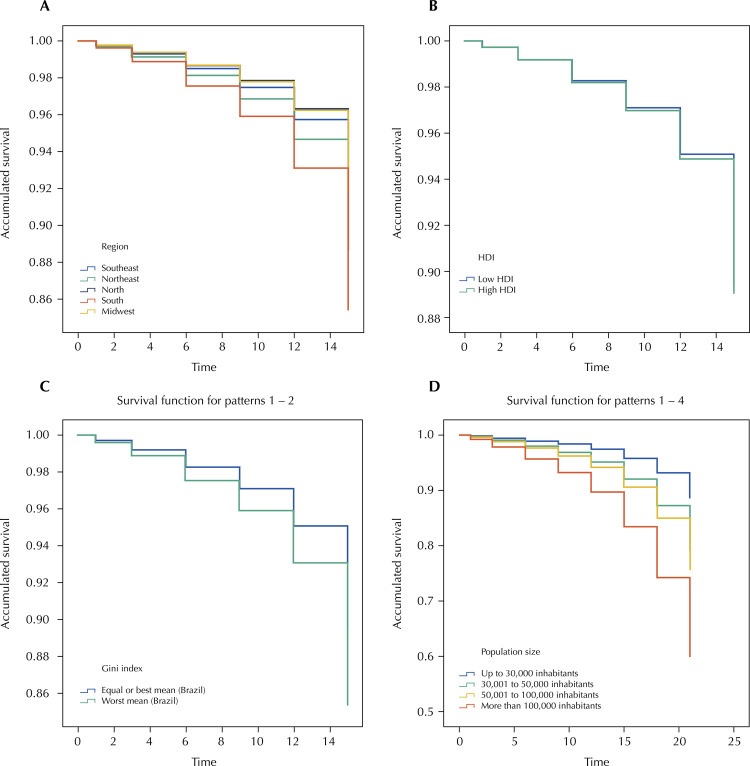
Cumulative survival of Brazilian municipalities that did not reduce the number of oral health teams from October 2017 to July 2019, according to the socioeconomic variables that comprised the Cox regression model. (A: region; B: HDI; C: Gini index; D: population size).

The region of the country, the Gini index and the population size were associated with a reduction in the number of OHT implanted in Brazil between October 2017 and July 2019. Municipalities in the Northeast (HR = 1.220) and South (HR = 1.771) regions had a higher chance of reducing the number of teams compared to those in the North region. Municipalities with higher Gini index (HR = 6.405) and with larger population size (HR = 4.273) were also more likely to reduce the number of OHT ( Table 2 ). Figure 3 shows the cumulative survival of Brazilian municipalities that did not reduce the number of OHT after the PNAB 2017, according to the socioeconomic variables that comprised the Cox regression model.

**Table 2:** Cox regression to verify the factors associated with the reduction in the number of oral health teams (OHT) in Brazil between October 2017 and July 2019.

	Regression coefficient	Error - standard	p	Hazard ratio	95% confidence interval
Region					
Southeast				1.000	
Northeast	0.199	0.078	0.011*	1.220	1.046–1.422
North	-0.073	0.107	0.496	0.930	0.755–1.146
South	0.572	0.058	< 0.001*	1.771	1.580–1.986
Midwest	-0.096	0.096	0.321	0.909	0.752–1.098
HDI	0.818	0.496	0.099	2.266	0.858–5.986
Gini Index	1.857	0.385	< 0.001*	6.405	3.013–13.614
Population size					
Up to 30,000 inhabitants				1.000	
30,001 to 50,000 inhabitants	0.656	0.068	< 0.001*	1.927	1.687–2.200
50,001 to 100,000 inhabitants	0.818	0.075	< 0.001*	2.265	1.956–2.623
More than 100,000 inhabitants	1.452	0.071	< 0.001*	4.273	3.716–4.914

HDI: human development index. * Statistically significant (p < 0.05).

HDI: human development index

## DISCUSSION

According to our results, the number of OHT implanted in Brazilian municipalities decreased after the PNAB 2017. There was a greater reduction in the municipalities located in the Northeast and South regions, with a higher Gini index and with a larger population size. This is in contrast to the history of construction of the National Oral Health Policy in the country, which brings the need for expansion of oral health in primary care in its guidelines ^7^ . Moreover, the evaluation period may be insufficient to verify significant effects of the PNAB 2017, which may reverse the trend of relative growth of oral health coverage in primary health care in Brazil ^6^ .

The number of municipalities that reduced the number of OHT after one year and nine months of publication of the PNAB 2017 tripled, reaching mainly those with the largest population in the South and Northeast regions. The impact in the South region may be more significant due to its low oral health coverage (36.2%), being ahead only of the Southeast region (27.2%). This phenomenon may represent a reduction in users’ access to oral health services, with effects on health conditions and quality of life ^15 , 16^ . In addition, the reduction of access to health is related to greater inequality in oral health, which is in line with the movement to expand the supply of services in primary care and reduce unfair inequalities ^17 , 18^ .

Although the Northeast region has the highest coverage in oral health among the regions of Brazil (66.3%), epidemiological data point to a greater need for oral health. The higher proportion of SUS-dependent population, higher epidemiological demand and lower socioeconomic status in the region are probably associated with greater coverage of care ^19 , 20^ . However, the reduction in the supply of dental services in PHC can negatively affect the region ^21 , 22^ . As observed in our study, the municipalities of the Northeast seem to be influenced by the new PNAB.

The reduction in the number of OHT among some Brazilian municipalities complements data from previous studies that show that the increase in the number of oral health teams in Brazil positively affects the use and access to dental services ^23 , 24^ , as well as greater user satisfaction ^25^ . A study conducted with 12-year-old schoolchildren and adolescents aged 15 to 19 years in Rio Grande do Sul showed that young people covered by the OHT have greater use of dental health services than those not covered ^26^ . Thus, the reduction in the number of OHT in the country is a problem that can aggravate health inequalities, as well as reduce the access of those that need dental services the most provided by SUS.

The human development index showed statistical association only in the bivariate model, and it was observed that municipalities classified as with high and very high HDI reduced the amount of OHT. Although municipalities with better HDI suggest a marked degree of quality of life for their populations, it does not mean that there are no social problems. One study identified a pro-equity trend regarding indicators of opportunity for access to oral health in PHC ^14^ . However, this trend is not yet reflected in the indicators of use of specialized services, in which the proportion of specialized dental procedures in relation to individual dental actions is higher among the federative units belonging to the quintile with the highest HDI. Although this phenomenon suggests a pro-equity trend, 68% of the Brazilian population are in the 35% of Brazilian municipalities with high or very high HDI; therefore, the reduction in the number of OHT will reach a large number of Brazilians.

The Gini index also showed a statistical association with the number of OHT implanted, and we observed that more unequal municipalities had a higher chance (HR = 6.405) of reducing the number of teams in PHC. Studies have shown that adolescents that lived during childhood in cities with income inequality are 1.75 times more likely to have oral impacts on daily performances (OIDP) ^27^ . In addition, the families with the lowest income have the greatest impact of dental health on daily activities ^28^ . Therefore, the reduction in the provision of dental health services in the most unequal places may negatively affect access to dental services, as well as individual and collective activities that stimulate preventive methods of dental injuries. The current economic political scenario points to the accentuation of inequities in health, which represents a gloomy picture for the Brazilian population dependent on the SUS.

The fact that municipalities with more than 100,000 inhabitants and consequently greater economic power are four times more likely to reduce the number of dental health professionals in PHC is intriguing. Although these locations concentrate most of the Brazilian population, the coverage of services is not broad. Consequently, the reduction in the supply of services will affect many people, especially those with worse socioeconomic conditions. This scenario is opposed to the beginning of the 2000s, strongly marked by the expansion of the FHS in large urban centers and the incorporation and expansion of OHT ^8 , 9^ , in response to the policies of promotion and qualification of PHC promoted by the National Primary Care Policy of the time and by *Brasil Sorridente* since 2004.

Although the studies do not confirm the impact of primary oral health coverage on the oral health condition of the population, the incorporation of OHT into the FHS seems more effective for the increase of indicators of use of dental services ^24^ . Moreover, the lower availability of dental health professionals may reduce the possibility of access to services. Finally, it may affect the epidemiological picture, given the historical process of dental health care in Brazil, characterized by low coverage, restricted access of the population to public dental health services and a disseminated practice of emergency services focused on extraction ^8 , 9^ .

If we understand dental health as something intrinsic and inseparable from human health, which cannot be disregarded when the objective is health care, the presence of dental health workers in PHC becomes essential, since this role is not fully performed by another member of the health team ^29^ . Due to the dental health conditions of Brazilians and the low coverage of dental health in the country, public policies are that strengthen *Brasil Sorridente* and the National Primary Care Policy are necessary, aiming at the integrality of care, to positively impact the expansion of supply and the qualification of the work process of dental health services in PHC.

Our findings may help researchers and managers in the evaluation of public policies directed to dental health in the Unified Health System. Other studies evaluating not only the effect on the implementation of oral health teams, but also the work process of the teams and the organization of the health care network are necessary to evaluate the impact of the PNAB on the provision of dental health services in the SUS.

Our evaluation was based on socioeconomic indicators and data available in information systems, which were evaluated under a multivariate statistical model. Although scientific research seeks to reduce the risk of bias, it is possible to assume that the characteristics associated with the reduction in the number of OHT are not necessarily identified in a single municipality or in a small set of them. In our study, we analyzed the factors associated with this investigation from the perspective of Brazil. Future evaluations should be conduct to confirm our findings.

Our study design limits the ability to identify the real effect of PNAB 2017 on the reduction of the number of teams. However, the findings of this investigation point to a stabilization of the total number of OHT. Moreover, the factors associated with the reduction in the number of teams point to the accentuation of social inequalities.

## CONCLUSION

Our study verified an increase in the number of municipalities that reduced the number of oral health teams in the Family Health Strategy over the period analyzed, especially in the South and Northeast regions, which have greater social inequality and larger population size. This scenario can significantly affect the population’s access to dental health services in the Unified Health System, especially among those most in need.
