# Supplementary material for: Monitoring of oral health teams after National Primary Care Policy 2017
Source: Rev Saude Publica. 2020 Oct 28;54:99. doi: 10.11606/s1518-8787.2020054002075 (PMC7593040; doi:10.11606/s1518-8787.2020054002075)
Supplement: Translation

# Translation: Monitoramento das equipes de saúde bucal após a Política Nacional de Atenção Básica 2017

**Authors:** Edson Hilan Gomes de Lucena, Carolina Dantas Rocha Xavier de Lucena, Josiane Aparecida de Souza Alemán, Gilberto Alfredo Pucca, Antônio Carlos Pereira, Yuri Wanderley Cavalcanti

**Affiliations:** I Brasil Universidade Federal da Paraíba. Centro de Ciências da Saúde. Departamento de Clínica e Odontologia Social. João Pessoa, PB, Brasil; II Brasil Fundação Oswaldo Cruz. Instituto Aggeu Magalhães. Programa de Pós-graduação em Saúde Pública. Pernambuco, PE, Brasil; III Brasil Universidade Federal da Paraíba. Centro de Ciências da Saúde. Núcleo de Estudo e Pesquisas Interdisciplinares em Biomateriais. João Pessoa, PB, Brasil; IV Brasil Universidade de Brasília. Faculdade de Ciências da Saúde. Departamento de Odontologia. Brasília, DF, Brasil; V Brasil Universidade Estadual de Campinas. Faculdade de Odontologia de Piracicaba. Departamento de Odontologia Social. Campinas, SP, Brasil

**Keywords:** Serviços de Saúde Bucal, provisão & distribução, Equipe de Assistência ao Paciente, Estratégia Saúde da Família, Disparidades nos Níveis de Saúde

## Abstract

**OBJETIVO:**

Realizar um monitoramento do quantitativo de equipes de saúde bucal implantadas na Estratégia Saúde da Família após a Política Nacional de Atenção Básica 2017.

**METODOLOGIA:**

Estudo de natureza quantitativa, descritiva e analítica que utilizou os dados dos relatórios públicos do histórico de cobertura de saúde bucal disponível na plataforma e-Gestor da Atenção Básica do Ministério da Saúde de todos os municípios brasileiros (5.570). A sobrevida dos municípios que não reduziram o quantitativo de equipes de saúde bucal foi analisada segundo a região do país, índice de desenvolvimento humano, índice de desigualdade de Gini e porte populacional. A regressão de Cox foi utilizada para analisar os fatores associados à diminuição do número de equipes implantadas após 1, 3, 6, 9, 12, 15, 18 e 21 meses da publicação da portaria da política nacional de 2017, considerando-se a *hazard ratio* (HR) e p < 0,05.

**RESULTADOS:**

Após 21 meses de publicação da política, 6,7% dos municípios brasileiros reduziram a quantidade de equipes de saúde bucal. Essa redução foi maior nas regiões Sul (6,7%) e Nordeste (4,8%), nos municípios com índice de desenvolvimento humano mais alto, ou seja, maior ou igual a 0,7 (5,6%), mais desiguais quanto à distribuição de renda (índice de Gini > 0,62) e de maior porte populacional (mais de 100.000 habitantes). Municípios das regiões Nordeste (HR = 1,220) e Sul (HR = 1,771) apresentaram maior chance de redução do número de equipes comparados aos da região Norte. Municípios mais desiguais (HR = 6,405) e com maior porte populacional (HR = 4,273) também apresentaram maior chance de reduzir a cobertura de equipes de saúde bucal.

**CONCLUSÃO:**

Os municípios que reduziram a quantidade de equipes de saúde bucal na Estratégia Saúde da Família são das regiões Sul e Nordeste, com maior desigualdade social e maior porte populacional. Esse cenário pode impactar significativamente o acesso da população aos serviços de saúde bucal do Sistema Único de Saúde, principalmente entre os que mais necessitam.

## INTRODUÇÃO

A atenção primária à saúde (APS) é o nível de assistência que desenvolve um conjunto de ações abrangendo desde a promoção à saúde, prevenção de doenças, proteção, diagnóstico e tratamento, bem como a reabilitação, redução de danos, cuidados paliativos e vigilância em saúde. Essas ações são direcionadas à população que está sob responsabilidade sanitária das equipes de saúde em determinado território, sendo desenvolvidas no âmbito individual, familiar e coletivo ^1^ .

No Brasil, as políticas de fortalecimento da APS são imprescindíveis para a efetivação dos princípios e diretrizes do Sistema Único de Saúde (SUS) ^2^ . Três importantes características fazem com que a organização da APS no SUS se diferencie de outros modelos propostos em diversos países: equipe multiprofissional responsável por determinados territórios geográficos com a população adscrita, presença dos agentes comunitários de saúde e inclusão da oferta integral da saúde bucal no sistema público de saúde ^3^ .

Historicamente, a inserção das práticas odontológicas no SUS ocorreu de forma paralela à organização dos demais serviços de saúde, com oferta de ações centradas na atenção materno-infantil e de escolares, sendo o enfoque preventivo restrito a esse grupo populacional. O perfil mutilador das ações de saúde bucal desenvolvidas até o início do século XXI resultou em elevada prevalência de indivíduos desdentados, o que estimulou a transformação das práticas em saúde bucal ^4^ . A incorporação da equipe de saúde bucal (ESB) em âmbito nacional, embora de forma opcional, ocorreu oito anos após a criação da Estratégia Saúde da Família (ESF) numa tentativa de reorganizar o modelo de oferta de cuidado em saúde bucal na APS ^5^ . Dessa forma, a inclusão da saúde bucal na ESF buscou romper com a prática odontológica excludente, tecnicista e biologicista, constituindo, portanto, oportunidade de mudança no processo de trabalho ^6^ .

A Política Nacional de Saúde Bucal (PNSB), Brasil Sorridente, implantada em 2004, estabeleceu diretrizes contextualizadas aos fundamentos do SUS, as quais reforçam a necessidade de reorganização da atenção à saúde bucal em todos os níveis de atenção. No âmbito da assistência, a PNSB aponta fundamentalmente para articulação da rede de saúde bucal de modo a buscar a integralidade da atenção ^7^ .

A partir do Brasil Sorridente, políticas foram implantadas visando a ampliação e qualificação da saúde bucal na APS, tais como os reajustes nos repasses financeiros, atualização da regra para implantação das ESB, equiparação da quantidade de ESB credenciadas às equipes de saúde da família, inclusão de novos procedimentos clínicos na APS e criação do plano de fornecimento de equipamentos odontológicos para cada ESB que o município viesse a implantar. Em 2012, a redefinição da composição das ESB na ESF foi uma das estratégias para consolidação da saúde bucal na APS ^8 , 9^ .

Os investimentos no Brasil Sorridente permitiram uma expansão de aproximadamente 500% no número de ESB, o que permitiu a ampliação de 4.261 ESB em 2002 para mais de 25.000 ESB em 2017, resultando em uma cobertura de cerca de 40% da população brasileira ^1^ . Embora tenham sido verificados avanços nos mais de 10 anos do Brasil Sorridente, o cenário político -econômico do Brasil sofreu transformações nos últimos anos. A publicação da Emenda Constitucional nº 95, que limitou o teto dos gastos com saúde e educação, terá um impacto significativo em longo prazo no total dos investimentos destinados ao setor, em descompasso com as demandas da população ^10^ . A portaria nº 2.436 do Ministério da Saúde, que reformulou a Política Nacional de Atenção Básica (PNAB) em 2017, prevê a não obrigatoriedade da saúde bucal na Estratégia Saúde da Família ^11^ . O risco de possíveis retrocessos, de desassistência de parte significativa da população e de perda na qualidade dos serviços da APS após a publicação dessa portaria foi apontado pela literatura ^2 , 12^ .

Nesse sentido, este estudo teve o objetivo de realizar um monitoramento do quantitativo de equipes de saúde bucal implantadas na Estratégia Saúde da Família após a Política Nacional de Atenção Básica 2017. Este trabalho faz uma análise da tendência do número de equipes implantadas e busca verificar quais fatores estão associados à redução do número de ESB no Brasil entre outubro de 2017 e julho de 2019.

## MÉTODOS

O presente estudo, de natureza quantitativa, descritiva e analítica, utilizou os dados disponibilizados nos relatórios públicos do histórico de cobertura de saúde bucal disponível na plataforma e-Gestor Atenção Básica do Ministério da Saúde, no período de outubro de 2017 a julho de 2019, de todos os 5.570 municípios brasileiros ^13^ .

A variável dependente (variação no número de ESB implantadas) foi categorizada da seguinte forma: “não alterou ou ampliou” e “reduziu” o número de equipes implantadas. As variáveis independentes do estudo foram: tempo – 1, 3, 6, 9, 12, 15, 18 e 21 meses após a publicação da nova P N A B ; região – Norte, Nordeste, Sudeste, Sul e Centro-Oeste; índice de desenvolvimento humano (IDH) – baixo (< 0,7) e alto (≥ 0,7); índice de Gini – menos desigual (≤ 0,61) e mais desigual (> 0,62); e porte populacional – até 30.000 habitantes, 30.001 a 50.000 habitantes, 50.001 a 100.000 habitantes e mais de 100.000 habitantes. O IDH e o índice de Gini foram obtidos pelo Censo Demográfico de 2010, divulgado pelo Programa das Nações Unidas para o Desenvolvimento (PNUD). Por esse motivo, o total de municípios para as variáveis IDH e Gini é de 5.565, correspondente ao número de municípios existentes em 2010. O porte populacional foi obtido do Instituto Brasileiro de Geografia e Estatística (IBGE).

Os dados foram inicialmente analisados por estatística descritiva visando caracterizar a amostra, sendo obtidas as distribuições absolutas e percentuais. Em seguida, realizou-se análise bivariada pelo teste do qui-quadrado de Pearson para a identificação de associações entre a variável dependente e as independentes.

Para a análise multivariada, realizou-se cálculo da sobrevida acumulada dos municípios brasileiros que não reduziram, isto é, que mantiveram ou aumentaram o número de ESB implantadas. Para tanto, utilizou-se a regressão de Cox para analisar os fatores associados à redução do número de ESB implantadas após 1, 3, 6, 9, 12, 15, 18 e 21 meses da publicação da portaria da nova PNAB. Os fatores região, IDH, índice de Gini e porte populacional foram utilizados como variáveis independentes que poderiam predizer a redução do número de ESB implantadas. Na análise de regressão, as variáveis IDH e Gini foram utilizadas como quantitativas contínuas, sem categorização.

Valores de *hazard ratio* (HR) foram obtidos para cada categoria dos fatores associados, considerando-se o intervalo de confiança de 95% e a significância estatística de 5%. Todas as tabulações e análises dos dados foram realizadas no software *Statistical Package for Social Sciences* (IBM-SPSS, v.24, IBM, Chicago, IL).

## RESULTADOS

De janeiro de 2017 a julho de 2019, o número de equipes de saúde bucal implantadas no Brasil foi de 25.848 para 28.311. No entanto, a linha de tendência indica uma estabilização no quantitativo dessas equipes após janeiro de 2018, indicando assim perda na amplitude de implantação ( Figura 1 ).

**Figura 1:** Número de equipes de saúde bucal da Estratégia Saúde da Família no Brasil entre janeiro de 2017 e julho de 2019.

jan: janeiro; fev: fevereiro; mar: março; abr: abril; mai: maio; jun: junho; jul: julho; ago: agosto; set: setembro; out: outubro; nov: novembro; dez: dezembro

Entre o 1º e o 21º mês da publicação da portaria da PNAB 2017, observou-se aumento de três vezes no número de municípios que reduziram a quantidade de ESB na ESF. Essa redução foi maior nas regiões Sul (6,7%) e Nordeste (4,8%), nos municípios com IDH mais alto (5,6%), mais desiguais quanto à distribuição de renda (índice de Gini > 0,62) e de maior porte populacional (mais de 100.000 habitantes). Pode-se observar que todas as variáveis apresentaram significância estatística no modelo bivariado ( Tabela 1 ). No entanto, não houve diferença estatisticamente significante (p = 0,081) entre o quantitativo de municípios que não alteraram ou ampliaram e os que reduziram o número de ESB no 18º e 21º mês, o que significa não haver uma tendência de redução no número de municípios que perderam equipes ( Tabela 1 ).

**Tabela 1:** Distribuição dos municípios brasileiros quanto à variação no número de equipes de saúde bucal (ESB) implantadas, segundo o tempo desde a publicação da p ortaria da nova Política Nacional de Atenção Básica, região do país, índice de desenvolvimento humano (IDH), índice de Gini e porte populacional.

		Variação no número de ESB implantadas				p
		Não reduziu		Reduziu		
		n	%	n	%	
Tempo	1 mês	5.457	98,0	113	2,0	< 0,001
	3 meses	5.398	96,9	172	3,1	
	6 meses	5.322	95,5	248	4,5	
	9 meses	5.333	95,7	237	4,3	
	12 meses	5.296	95,1	274	4,9	
	15 meses	5.187	93,1	383	6,9	
	18 meses	5.156	92,6	414	7,4	
	21 meses	5.203	93,4	367	6,6	
Região	Norte	430	95,5	20	4,5	< 0,001
	Nordeste	1.708	95,2	86	4,8	
	Sudeste	1.595	95,6	73	4,4	
	Sul	1.111	93,3	80	6,7	
	Centro-Oeste	450	96,4	17	3,6	
IDH	Baixo (< 0,7)	3.502	96,4	130	3,6	< 0,001
	Alto (≥ 0,7)	1.826	94,4	107	5,6	
Índice de Gini	Menos desigual (≤ 0,61)	5.133	95,8	225	4,2	0,010
	Mais desigual (> 0,62)	195	94,3	12	5,7	
Porte populacional	Até 30.000 habitantes	4.251	96,4	160	3,6	< 0,001
	30.001 a 50.000 habitantes	458	92,9	35	7,1	
	50.001 a 100.000 habitantes	319	91,4	30	8,6	
	Mais de 100.000 habitantes	266	83,8	52	16,2	

Verificou-se que 6,6% (n = 367) dos municípios brasileiros reduziram o número de ESB na Estratégia Saúde da Família após 15 meses da publicação da PNAB em 2017. A curva de sobrevida dos municípios que não reduziram o quantitativo de ESB demonstrou maior redução entre novembro 2018 e julho 2019 ( Figura 2 ). Maior redução foi verificada no mês de abril de 2019 (18º mês).

**Figura 2:** Sobrevida acumulada dos municípios brasileiros que não reduziram o número de equipes de saúde bucal entre outubro de 2017 e julho de 2019.

A região do país, o índice de Gini e o porte populacional foram associados à redução do número de ESB implantadas no Brasil entre outubro de 2017 e julho de 2019. Municípios das regiões Nordeste (HR = 1,220) e Sul (HR = 1,771) apresentaram maior chance de redução do número de equipes comparados aos da região Norte. Municípios com maior índice de Gini (HR = 6,405) e com maior porte populacional (HR = 4,273) também apresentaram maior chance de reduzir o quantitativo de ESB ( Tabela 2 ). A Figura 3 ilustra a sobrevida acumulada dos municípios brasileiros que não reduziram o número de ESB após a PNAB 2017, segundo as variáveis socioeconômicas que compuseram o modelo de regressão de Cox .

**Tabela 2:** Regressão de Cox para verificar os fatores associados à redução do número de equipes de saúde bucal (ESB) no Brasil entre outubro de 2017 e julho de 2019.

	Coeficiente de regressão	Erro-padrão	p	Hazard ratio	Intervalo de confiança de 95%
Região					
Sudeste				1,000	
Nordeste	0,199	0,078	0,011*	1,220	1,046–1,422
Norte	-0,073	0,107	0,496	0,930	0,755–1,146
Sul	0,572	0,058	< 0,001*	1,771	1,580–1,986
Centro-Oeste	-0,096	0,096	0,321	0,909	0,752–1,098
IDH	0,818	0,496	0,099	2,266	0,858–5,986
Índice de Gini	1,857	0,385	< 0,001*	6,405	3,013–13,614
Porte populacional					
Até 30.000 habitantes				1,000	
30.001 a 50.000 habitantes	0,656	0,068	< 0,001*	1,927	1,687–2,200
50.001 a 100.000 habitantes	0,818	0,075	< 0,001*	2,265	1,956–2,623
Mais de 100.000 habitantes	1,452	0,071	< 0,001*	4,273	3,716–4,914

* Estatisticamente significante (p < 0,05).

**Figura 3:** Sobrevida acumulada dos municípios brasileiros que não reduziram o número de equipes de saúde bucal entre outubro de 2017 e julho de 2019, segundo as variáveis socioeconômicas que compuseram o modelo de regressão de Cox (A: região; B: IDH; C: índice de Gini; D: porte populacional).

IDH: índice de desenvolvimento humano

## DISCUSSÃO

De acordo com os resultados deste estudo, observou-se que o quantitativo de ESB implantadas nos municípios brasileiros reduziu após a PNAB 2017. Verificou-se maior redução nos municípios localizados nas regiões Nordeste e Sul, com maior índice de Gini e com maior porte populacional. Isso se contrapõe ao histórico de construção da Política Nacional de Saúde Bucal no país, a qual traz em suas diretrizes a necessidade de expansão da saúde bucal na atenção básica ^7^ . Além disso, o período de avaliação pode ser insuficiente para verificar efeitos significativos da PNAB 2017, que pode reverter a tendência de crescimento relativo da cobertura de saúde bucal na atenção primária à saúde no Brasil ^6^ .

O número de municípios que reduziram a quantidade de ESB após um ano e nove meses da publicação da PNAB 2017 triplicou, atingindo principalmente os de maior porte populacional nas regiões Sul e Nordeste. O impacto na região Sul pode ser mais significativo pois essa região possui baixa cobertura de saúde bucal (36,2%), ficando à frente apenas da região Sudeste (27,2%). Esse fenômeno pode representar uma redução no acesso dos usuários aos serviços de saúde bucal, com consequentes impactos nas condições de saúde e qualidade de vida ^15 , 16^ . Além disso, a redução do acesso em saúde está relacionada à maior iniquidade em saúde bucal, o que vai de encontro ao movimento de ampliação da oferta de serviços na atenção primária e redução de desigualdades injustas ^17 , 18^ .

Embora a região Nordeste apresente a maior cobertura em saúde bucal entre as regiões do Brasil (66,3%), os dados epidemiológicos apontam para maior necessidade em saúde bucal. A maior proporção de população dependente do SUS, maior demanda epidemiológica e menor condição socioeconômica da região est ão provavelmente associadas à maior cobertura da assistência ^19 , 20^ . Entretanto, a redução da oferta de serviços odontológicos na APS pode impactar negativamente a região ^21 , 22^ . Conforme observado neste estudo, os municípios do Nordeste parecem sofrer influência da nova PNAB.

A redução do número de ESB entre alguns municípios brasileiros complementa dados da literatura que demonstram que a ampliação do número de equipes de saúde bucal no Brasil impacta positivamente o uso e o acesso aos serviços odontológicos ^23 , 24^ , bem como a maior satisfação dos usuários ^25^ . Um estudo realizado com escolares de 12 anos e adolescentes de 15 a 19 anos no Rio Grande do Sul demonstrou que os jovens cobertos pela ESB apresentam maior uso dos serviços de saúde bucal do que os não cobertos ^26^ . Sendo assim, a redução do número de ESB no país constitui um problema que pode agravar as desigualdades em saúde, bem como reduzir o acesso daqueles que mais necessitam dos serviços odontológicos na perspectiva do SUS.

O índice de desenvolvimento humano apresentou associação estatística apenas no modelo bivariado, sendo observado que municípios classificados com IDH elevado e muito elevado reduziram a quantidade de ESB. Embora os municípios com melhor IDH sugiram um acentuado grau de qualidade de vida de suas populações, não significa que não existam problemas sociais. Um estudo identificou tendência pró-equidade no que diz respeito aos indicadores de oportunidade de acesso na saúde bucal da APS ^14^ . Entretanto, essa tendência ainda não se reflete nos indicadores de uso dos serviços especializados, nos quais a proporção de procedimentos odontológicos especializados em relação às ações odontológicas individuais é maior entre as unidades federativas pertencentes ao quintil de maior IDH. Embora esse fenômeno sugira tendência pró-equidade, 68% da população brasileira encontram-se nos 35% dos municípios brasileiros que apresentam o IDH elevado ou muito elevado, portanto, a redução do número de ESB atingirá uma grande quantidade de brasileiros.

O índice de Gini também apresentou associação estatística com o número de ESB implantadas, sendo observado que municípios mais desiguais tiveram maior chance (HR = 6,405) de reduzir o quantitativo de equipes na APS. Estudos têm demonstrado que os adolescentes que moraram durante a infância em cidades com desigualdade de renda tê m 1,75 vezes mais probabilidade de ter impacto odontológico no desempenho diário ( *Oral Impacts on Daily Performances* – OIDP) ^27^ . Além disso, as famílias de menor renda são as que tiveram maior impacto da saúde bucal nas atividades diárias ^28^ . Portanto, a redução na oferta de serviços de saúde bucal nos locais mais desiguais poderá impactar de forma negativa o acesso aos serviços odontológicos, bem como atividades individuais e coletivas que estimulem os métodos preventivos de agravos bucais. O cenário político econômico atual aponta para a acentuação das desigualdades e das iniquidades em saúde, o que representa um quadro sombrio para população brasileira dependente do SUS.

O fato de os municípios com mais de 100.000 habitantes e consequente maior poder econômico apresentarem quatro vezes mais chance de reduzir a quantidade de profissionais de saúde bucal na APS é intrigante. Embora esses locais concentrem maior parte da população brasileira, a cobertura de serviços não é ampla. Consequentemente, a redução da oferta de serviços atingirá uma grande quantidade de pessoas, em especial aquelas com piores condições socioeconômicas. Esse cenário se opõe ao início dos anos 2000, fortemente marcados pela expansão da ESF nos grandes centros urbanos e pela incorporação e ampliação das ESB ^8 , 9^ , em resposta às políticas de fomento e qualificação da APS promovidas pela Política Nacional de Atenção Básica da época e pelo Brasil Sorridente a partir de 2004.

Muito embora os estudos não confirmem o impacto da cobertura da atenção básica em saúde bucal na condição de saúde bucal da população, há de se ponderar que a incorporação de ESB à ESF parece mais efetiva para o aumento de indicadores de uso de serviços odontológicos ^24^ . Além disso, a menor disponibilidade de profissionais de saúde bucal poderá reduzir a possibilidade de acesso aos serviços. Em última instância, poderá impactar o quadro epidemiológico, haja vista o processo histórico do cuidado de saúde bucal no Brasil, caracterizado pela baixa cobertura, acesso restrito da população aos serviços públicos de saúde bucal e prática disseminada de serviços de urgência com enfoque na exodontia ^8 , 9^ .

Com a compreensão da saúde bucal como algo intrínseco e indissociável da saúde humana, a qual não pode ser desconsiderada quando o objetivo é o cuidado, torna-se fundamental a presença de trabalhadores da saúde bucal na APS, visto que esse papel não é exercido de forma integral por outro membro da equipe de saúde ^29^ . Tendo em vista as condições de saúde bucal dos brasileiros e a baixa cobertura de saúde bucal no país, fazem-se necessárias políticas públicas que fortaleçam o Brasil Sorridente e a Política Nacional de Atenção Básica, visando a integralidade do cuidado, de modo a impactar positivamente a ampliação da oferta e a qualificação do processo de trabalho dos serviços de saúde bucal na APS.

Os achados deste estudo podem contribuir com pesquisadores e gestores na avaliação das políticas públicas direcionadas para a saúde bucal no Sistema Único de Saúde. Outros estudos que avaliem não só o efeito na implantação das equipes de saúde bucal, mas também o processo de trabalho das equipes e a organização da rede de atenção à s aúde b ucal são necessários para avaliar o impacto da PNAB na oferta de serviços de saúde bucal no SUS.

Destaca-se que esta avaliação se baseou em indicadores socioeconômicos e dados disponíveis em sistemas de informação, os quais foram avaliados sob um modelo estatístico multivariado. Embora a pesquisa científica busque reduzir o risco de viés, é possível assumir que as características associadas à redução do número de ESB não são necessariamente identificadas em um único município ou em um pequeno conjunto deles. É importante destacar que os fatores associados nesta investigação foram analisados sob a perspectiva do Brasil. Avaliações futuras devem ser realizadas para confirmar os achados desta pesquisa.

O delineamento deste estudo limita a capacidade de identificar o real efeito da PNAB 2017 na redução do quantitativo de equipes. Entretanto, os achados desta investigação apontam para uma estabilização do quantitativo total do número de ESB. Além disso, os fatores associados à redução do número de equipes apontam para a acentuação das desigualdades sociais.

## CONCLUSÃO

O presente estudo verificou, ao longo do período analisado, um aumento no número de municípios que reduziram a quantidade de equipes de saúde bucal na Estratégia Saúde da Família, especialmente n as regiões Sul e Nordeste, que apresentam maior desigualdade social e maior porte populacional. Esse cenário pode impactar significativamente o acesso da população aos serviços de saúde bucal do SUS, principalmente entre os que mais necessitam deles.
